# Novel Functions and Potential of Ribosomes: From Cellular Transdifferentiation to Applications in Cell-Cultured Foods

**DOI:** 10.3390/jdb14020017

**Published:** 2026-04-09

**Authors:** Shota Inoue, Hiroaki Hatano, Ikko Kawashima, Kunimasa Ohta

**Affiliations:** 1 Department of Stem Cell Biology, Graduate School of Systems Life Sciences, Kyushu University, 744 Motooka, Nishi-Ku, Fukuoka 819-0395, Japan; inoue.shota.090@s.kyushu-u.ac.jp; 2 IntegriCulture, 8-1 Kawada-Cho, Shinjuku-Ku, Tokyo 162-0054, Japan; hiroaki.hatano@integriculture.com (H.H.); ikko@integriculture.com (I.K.); 3 Department of Stem Cell Biology, Faculty of Arts and Science, Kyushu University, 744 Motooka, Nishi-Ku, Fukuoka 819-0395, Japan

**Keywords:** ribosomes, transdifferentiation, chick primary cells, cell proliferation, cell-cultured food

## Abstract

Ribosomes are widely recognized as large intracellular macromolecular complexes responsible for protein synthesis. However, in recent years, numerous studies have revealed that ribosomal proteins possess non-canonical functions beyond translation, including roles in cell fate regulation, development, and disease. This review outlines emerging concepts surrounding the extracellular functions of ribosomes, with a particular focus on ribosome-induced cellular plasticity and transdifferentiation. Our studies have demonstrated that the incorporation of exogenous ribosomes reprograms somatic cells into a multipotent state and promotes differentiation into multiple lineages. These findings represent an alternative perspective to the conventional view of ribosomes as merely translational components. Furthermore, we discuss the biological significance of factors secreted by ribosome-incorporated cells by integrating the paracrine hypothesis with ribosome-mediated cell fate conversion. Finally, we explore the potential applications of ribosomes in regenerative medicine and cell-cultured food production. By redefining ribosomes as active regulators of cellular identity, this review provides a conceptual framework for understanding ribosome-driven cell fate regulation and its potential applications in sustainable biotechnology.

## 1. Basic Structure and Function of Ribosomes

Ribosomes are large intracellular macromolecular complexes universally conserved across almost all forms of life and are classically recognized as the cellular machinery responsible for protein synthesis. Structurally, ribosomes are composed of a small and a large subunit, each consisting of multiple ribosomal proteins and ribosomal RNAs (rRNAs) [1]. Ribosomes translate genetic information encoded in messenger RNA (mRNA) into polypeptides by sequentially incorporating amino acids. Although this fundamental role is conserved, ribosomal structure and regulatory features differ substantially between prokaryotes and eukaryotes. In prokaryotes, such as bacteria, ribosomes are composed of a 30S small subunit and a 50S large subunit, forming a functional 70S ribosome [2,3,4]. In contrast, eukaryotic ribosomes are larger and structurally more complex, consisting of a 40S small subunit and a 60S large subunit that together form the 80S ribosome [5,6,7,8]. While eukaryotic ribosomes share an evolutionarily conserved core architecture with prokaryotic ribosomes, they possess additional structural elements and regulatory layers that enable sophisticated translational control, including context-dependent regulation and localized translation [9,10,11,12]. Thus, although prokaryotic and eukaryotic ribosomes share common principles, they exhibit functional diversity adapted to distinct cellular and organismal contexts [1,13].

Despite the highly conserved nature of ribosomes, advances in structural biology since the early 2000s have revealed their detailed architecture. High-resolution analyses using X-ray crystallography and cryo-electron microscopy have provided unprecedented insights into ribosomal three-dimensional structure and the molecular mechanisms underlying translation [1,14,15,16,17]. These studies have elucidated, at the molecular level, how mRNA decoding, tRNA translocation, and peptide bond formation proceed in a coordinated manner, establishing that the ribosome is not a static “protein synthesis machine” but a dynamic and precisely regulated molecular apparatus.

Accordingly, ribosomes are increasingly being re-evaluated as multifaceted entities that extend beyond their canonical role in translation to influence cellular states and fate decisions. Continued methodological advances are expected to further expand our understanding of ribosome-mediated regulation and its broader implications in life science.

## 2. Non-Canonical Functions of Ribosomes

Traditionally, ribosomes have been understood primarily as the translational machinery responsible for protein synthesis. However, recent studies have suggested that ribosomes and their constituent components may possess a variety of biological functions beyond translation. In 2012, Xue and colleagues proposed the specialized ribosome hypothesis [18], which was reviewed last year by Beavan et al. [19], emphasizing the now well-established heterogeneity in ribosome composition, but noting that the functional consequences of the structural variation in ribosomes remain poorly understood. According to this hypothesis, ribosomes are not uniform translational machines; rather, functionally distinct ribosome populations may exist due to variations in ribosomal protein composition and rRNA modifications, each potentially regulating the translation of particular subsets of mRNAs. In addition, the ribosome-associated stress response, in which translational abnormalities or perturbations in ribosome biogenesis trigger cellular stress responses, has also been reported [20,21,22]. These findings suggest that ribosomes may function as sensors that monitor cellular states. In this section, we first provide an overview of these concepts and then focus specifically on two aspects: the moonlighting functions of ribosomal proteins and cell state regulation mediated by exogenous ribosomes.

### 2.1. Moonlighting Functions of Ribosomal Proteins

Among intracellular proteins, some possess functions distinct from their originally recognized primary roles. Such proteins are referred to as moonlighting proteins, defined as single polypeptides capable of performing multiple functions depending on the cellular environment or conditions [23,24]. Ribosomal proteins are not exceptions to this concept, and accumulating evidence has revealed that many ribosomal proteins exert extra-ribosomal functions independent of their canonical roles in translation [25,26,27,28].

A representative example was reported by Vousden and colleagues, who demonstrated that the ribosomal protein L11 (RPL11) is involved in the regulation of the tumor suppressor p53 independently of translation [29]. RPL11 directly interacts with the E3 ubiquitin ligase HDM2, a major negative regulator of p53, thereby inhibiting HDM2-mediated ubiquitination and degradation of p53. Through this interaction, RPL11 stabilizes and activates p53. Notably, when ribosome biogenesis is perturbed—for example, through inhibition of RNA polymerase I activity—the interaction between RPL11 and HDM2 is enhanced, leading to p53-dependent cell cycle arrest. This study provided the first clear evidence that ribosomal proteins can function not merely as structural components of the translational machinery but also as signaling molecules that sense disruptions in ribosome biogenesis and transmit signals to regulate cell proliferation. Consequently, this work is widely regarded as one of the earliest representative examples of extra-ribosomal functions of ribosomal proteins. Subsequent studies further demonstrated that, in addition to RPL11, other ribosomal components—including RPL5 and precursor ribosomal complexes containing 5S rRNA—also participate in regulating the MDM2–p53 pathway [30]. These findings have led to the growing recognition that ribosomal components themselves can function as regulatory hubs in cellular stress responses.

As an example of extra-ribosomal functions in developmental regulation, Wiest and colleagues reported that Rpl22 and its paralog Rpl22l1 regulate morphogenesis during embryonic development independently of their ribosomal roles [31]. Although neither Rpl22 nor Rpl22l1 is essential for general protein synthesis, both are abundantly expressed from the two-cell stage in zebrafish embryos [32]. The authors demonstrated that these two proteins function antagonistically to regulate germ layer formation by modulating the splicing of pre-mRNA encoding Smad2, an essential transcriptional effector of the Nodal/TGF-β signaling pathway. This study provided an important example demonstrating that ribosomal proteins can directly participate in RNA processing and developmental fate determination, extending their roles beyond translational control.

Another representative example of the moonlighting functions of ribosomal proteins is the non-integrin 37/67 kDa laminin receptor (LAMR). LAMR was originally identified as a 67 kDa cell surface receptor that binds the extracellular matrix component laminin [33,34,35]. However, subsequent studies revealed that this receptor corresponds to a 37 kDa ribosomal protein, RPSA, which serves as its precursor [36]. While RPSA functions as a ribosomal component involved in protein synthesis, it also acts as a laminin receptor at the cell surface. Through this role, it has been implicated in a wide range of biological processes, including cell adhesion, cell migration, cell proliferation, developmental processes, and susceptibility to viral and bacterial infections [37].

Taken together, accumulating evidence indicates that many ribosomal proteins perform functions independently of translation. Examples include RPL11-mediated regulation of the p53 pathway, developmental regulation by Rpl22/Rpl22l1, and cell surface receptor functions represented by RPSA/LAMR. Dysregulation of these moonlighting ribosomal proteins has been implicated in various pathological conditions, including cancer, inflammatory diseases, ribosomopathies, and viral infections, highlighting the importance of viewing ribosomal components as multifunctional regulators of cellular processes [38]. More recently, this concept has begun to extend beyond intracellular ribosomal proteins to the possibility that exogenous ribosomes may influence cellular states. In the following section, we discuss studies describing cell state changes induced by exogenous ribosomes.

### 2.2. Exogenous Ribosomes and Cell State Changes

**Figure 1:**
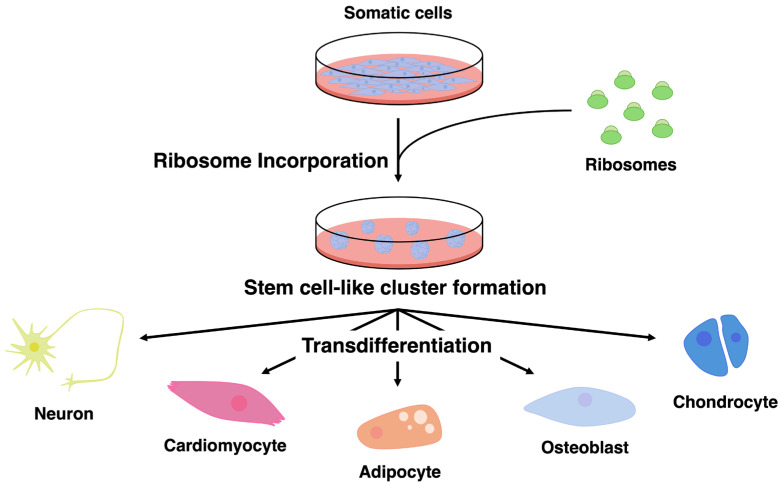
Ribosome incorporation induces stem cell-like cluster formation and multilineage transdifferentiation of somatic cells. Somatic cells incorporate exogenous ribosomes, leading to the formation of stem cell-like cell clusters characterized by increased cellular plasticity. These ribosome-incorporated clusters subsequently exhibit multilineage differentiation potential and can give rise to diverse cell types, including neurons, cardiomyocytes, adipocytes, osteoblasts, and chondrocytes, under appropriate lineage-specific induction conditions. This process occurs without genetic modification and suggests that ribosome incorporation reconfigures the cellular state of somatic cells, enabling broad transdifferentiation capacity.

Recent studies suggest that exogenous ribosomes may influence cellular states. Ohta and colleagues reported that the introduction of purified bacteria-derived ribosomes into somatic cells induces the formation of cell clusters exhibiting stem cell-like properties [39]. These clusters display altered gene expression patterns and multilineage differentiation potential, suggesting that exogenous ribosomes may contribute to cellular state changes (Figure 1). In their earlier studies, the authors reported that the incorporation of lactic acid bacteria into mammalian somatic cells induces cellular transformation and the acquisition of differentiation potential [40]. Subsequent work from the same group identified ribosomes as the causative molecular factor underlying this phenomenon [39,41]. More recently, similar phenomena have also been reported in cancer cells and avian cells, suggesting that this effect may not be restricted to a single cell type [42,43,44,45,46,47]. However, most of the current knowledge regarding cell state changes induced by exogenous ribosomes is primarily derived from studies conducted by the Ohta research group. Therefore, further investigations by independent research groups will be necessary to determine the generality and reproducibility of this phenomenon.

The molecular mechanisms underlying this process remain largely unclear. Studies from the same group have reported that once internalized, exogenous ribosomes localize to both the cytoplasm and the nucleus [39]. However, how these ribosomes influence intracellular signaling pathways or transcriptional regulation remains to be elucidated. Several hypotheses have been proposed [43]. One possibility is that ribosome uptake triggers cellular stress responses or signaling pathways, thereby altering gene expression programs. Another possibility is that ribosome-derived RNAs or ribosomal proteins may influence the epigenetic state of cells, leading to changes in transcriptional regulation. Furthermore, recent studies from the same group have suggested that ribosome-incorporated cells may release secreted factors containing growth factors that promote the proliferation of surrounding cells, raising the possibility that changes in paracrine signaling networks may also contribute to the observed cellular responses [47].

Interestingly, phenomena resembling host cell reprogramming have also been reported in the context of bacterial infection. For example, Rambukkana and colleagues reported that infection with *Mycobacterium leprae* induces the dedifferentiation of Schwann cells, converting them into a progenitor-like state [48]. This study is widely recognized as a representative example demonstrating that bacterial-derived factors can alter the cell fate of host cells. In subsequent studies, the same group used a natural infection model in nine-banded armadillos and reported that *M. leprae* infection promotes growth of the adult liver [49]. However, the specific bacterial-derived molecules responsible for altering host cell properties in these studies have not yet been identified, and further molecular-level investigations will be required to elucidate the underlying mechanisms.

Similarly, in plant–bacterial pathogen interactions, examples have been reported in which bacterial-derived molecules reprogram host transcriptional programs. For instance, transcription activator-like (TAL) effectors secreted by bacteria of the genus *Xanthomonas* are injected into host cells, translocate into the nucleus, and directly bind to host DNA, thereby reprogramming gene expression [50]. TAL effectors function as transcriptional activators and are known to promote the infection process by manipulating host transcriptional networks. Although the underlying molecular mechanisms differ, these studies collectively demonstrate that bacterial-derived molecules can alter host cellular states through diverse molecular strategies.

Another important issue concerns the relationship between intracellular ribosomes, extracellular ribosome-associated molecules, and extracellular vesicles (EVs). Recent studies have revealed that ribosomal RNA fragments and ribosomal proteins can be detected in the extracellular environment, where they constitute components of the extracellular RNAome. In particular, Cayota and colleagues reported that extracellular ribosomal RNAs can be found not only within extracellular vesicles but also as RNA–protein complexes independent of vesicular structures [51]. These findings highlight the importance of distinguishing extracellular ribosome-related molecules from EV-derived RNAs. At the same time, in studies using exogenous ribosomes, it is necessary to consider the possibility that the observed cellular responses may arise from other cellular components, such as EVs, rather than ribosomes themselves [52]. In the studies conducted by Ohta and colleagues, ribosomes were purified using an *Escherichia coli* strain (JE28), in which one of the ribosomal proteins was engineered to contain a His tag, allowing the ribosome complex to be isolated through Ni-affinity purification [53]. This approach specifically targets ribosomal proteins and therefore substantially reduces contamination by other cellular components. Through this purification strategy, extracellular vesicles and other cellular constituents are expected to be largely removed during the purification process, suggesting that the cellular state changes observed in these studies are most likely attributable to the purified ribosomal complexes themselves.

Taken together, these findings suggest that ribosomes may function not only as translational machinery, as traditionally understood, but also as novel regulatory factors capable of influencing cellular state changes. However, the number of independent studies addressing this phenomenon remains limited, and the underlying molecular mechanisms are still insufficiently understood. Therefore, further studies will be required to clarify the physiological significance of ribosome-mediated cellular reprogramming, as well as its potential applications.

## 3. Stem Cell-Derived Conditioned Media and the Paracrine Hypothesis

In regenerative medicine research using stem cells, increasing attention has recently been directed toward conditioned medium containing factors secreted by stem cells during culture, rather than the transplanted cells themselves. Traditionally, the therapeutic effects of stem cell transplantation were attributed to engraftment and differentiation of transplanted cells, leading to direct replacement of damaged tissues. However, in many tissues, including the myocardium and nervous system, functional improvement has been observed despite extremely low engraftment efficiency, giving rise to an apparent discrepancy [54]. To explain this phenomenon, the paracrine hypothesis was proposed, positing that the therapeutic effects of stem cells are mediated primarily by secreted factors [55,56]. According to this hypothesis, the principal contribution of stem cells lies not in their differentiation capacity per se, but in indirect actions mediated by growth factors, cytokines, chemokines, and EVs present in the conditioned medium. In particular, conditioned medium derived from mesenchymal stem cells exerts anti-apoptotic, pro-angiogenic, and anti-inflammatory effects in cardiovascular disease models [55,57]. These observations indicate that stem cell-conditioned medium represents a functional entity responsible for regenerative outcomes rather than a mere byproduct of cell culture.

A particularly important feature of stem cell-conditioned medium is that its biological activity arises from the coordinated action of multiple factors rather than from a single bioactive molecule. Conditioned media derived from mesenchymal stem cells or induced pluripotent stem cells contain a wide range of growth factors, including VEGF, HGF, IGFs, members of the FGF family, and PDGFs, each contributing distinct biological functions. Consequently, the activity of conditioned medium is widely recognized as a “cocktail effect,” in which biological outcomes depend on the combination and relative balance of factors rather than on individual components [56,58]. This concept emphasizes that the effects of stem cell-conditioned medium cannot be explained by a single signaling pathway, but instead emerge from the simultaneous and interconnected regulation of multiple signaling cascades. Indeed, regeneration-related responses such as cell proliferation, angiogenesis, anti-inflammatory effects, and suppression of cell death are not independent phenomena, but are efficiently induced only when multiple factors act in concert. This multi-pathway, cooperative mode of action constitutes a fundamental basis for the regenerative therapeutic value of stem cell-conditioned medium.

More recently, EVs—particularly exosomes—have attracted attention as key mediators of paracrine signaling. EVs encapsulate diverse molecular cargos, including proteins, lipids, and microRNAs, and modulate multiple signaling pathways upon uptake by recipient cells. Numerous studies have reported regenerative effects of MSC- and iPSC-derived EVs in diverse systems, including skin, neural tissues, and spinal cord injury models [59,60,61,62]. At the same time, increasing emphasis on EVs has led to interpretations that attribute the activity of conditioned medium primarily to EVs alone. However, the persistence of biological activity in EV-depleted conditioned medium, together with evidence suggesting functional interactions between EVs and soluble factors, indicates that the essence of stem cell-conditioned medium lies in its nature as a composite cocktail in which soluble factors and EVs coexist and act cooperatively [56,58,62].

Most previous studies have discussed paracrine effects under the implicit assumption that high secretory activity is an intrinsic property of stem cells. In other words, mesenchymal stem cells and induced pluripotent stem cells have been regarded as inherently high secretors, and the functions of their conditioned media have been interpreted as cell type-specific traits. However, we reported findings that prompt reconsideration of this assumption. Specifically, we demonstrated that incorporation of exogenous ribosomes induces not only cell fate plasticity but also stem cell-like secretory properties in primary chicken-derived somatic cells that are not stem cells [47]. Conditioned medium derived from these cells significantly enhances cell proliferation, an effect fundamentally comparable to the paracrine activity reported for mesenchymal stem cell- or induced pluripotent stem cell-derived conditioned media. Furthermore, transcriptomic analyses revealed coordinated upregulation of multiple growth factor-related genes, including PDGFB and members of the FGF and IGF families, demonstrating that a canonical cocktail effect can be reproduced without the use of stem cells. These findings extend the conventional framework in which the function of conditioned medium is viewed as an intrinsic property of stem cells and instead suggest that paracrine capacity is determined by cellular state rather than by cell identity. In this view, the high secretory activity of stem cells reflects a cellular state characterized by undifferentiation and plasticity, which activates secretory networks, rather than stemness as a categorical identity.

Taken together, stem cell-conditioned medium should be regarded not as a culture byproduct but as a functional, multi-factorial cocktail that mediates regenerative effects through coordinated, multi-pathway regulation. Notably, our findings integrate and extend existing knowledge by expanding the concept of conditioned-medium function beyond stem cell-restricted contexts.

## 4. Applications of Ribosome-Based Technologies in Cell-Cultured Food Production

Cell-cultured food (cultured meat) is food produced by cultivating cells derived from mammals, fish, and other sources in hygienically controlled in vitro [63,64,65]. In recent years, cell-cultured food has seen successive approvals for manufacturing in Singapore, the United States, and Israel, with commercialization progressing.

One current development trend in cell-based food production is reducing manufacturing costs. Efforts to lower these costs include developing low-cost serum-free media, switching from reagents to ingredients with a history of food use, and developing bioreactors. Various companies are advancing development aimed at cost reduction [66,67,68]. Another development trend in cell-cultured foods is the advancement of product development utilizing diverse cell types and animal species. For instance, companies like Upside Foods and GOOD Meat are developing products using chicken-derived cells [69,70]. Wildtype is advancing development using salmon-derived cells, while Mission Barns employs pig-derived cells [71,72]. Examples thus demonstrate the utilization of cells sourced from birds, fish, and mammals. An interesting example is Mission Barns’ porcine adipose cells. Adipose cells have been shown to significantly contribute to texture and flavor in processed foods, demonstrating efforts to leverage the unique qualities (taste and nutritional value) inherent in animal cells [73]. Furthermore, as an example utilizing uncommon poultry, Vow, approved in Australia and New Zealand, produces quail-derived cells, creating novel experiences for consumers [74]. This diversification of cell-cultured foods is noteworthy as an attempt to penetrate the market by exploring and proposing products that society demands. Efforts to launch new products into the market are expected to continue, driving progress in creating cell types derived from various animals. Furthermore, to produce diverse cell types, there is a corresponding need to standardize cell quality. As cell-cultured foods are intended for food applications, technologies using food ingredients to stabilize cell quality are desirable.

**Figure 2:**
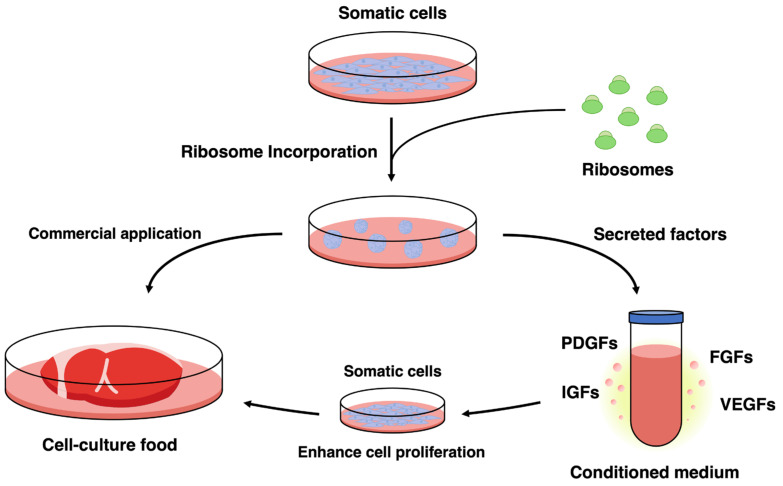
Ribosome-induced cellular states enable both cell-cultured food production and cell-free applications through secreted factors. Incorporation of exogenous ribosomes into somatic cells induces a stem cell-like cellular state that supports both differentiation-based and secretion-based applications. Ribosome-incorporated cells can be directly utilized for cell-cultured food production, representing a potential commercial application. In parallel, these cells secrete multiple bioactive factors, including PDGFs, FGFs, IGFs, and VEGFs, which are released into the conditioned medium. The conditioned medium enhances the proliferation of somatic cells without the need for exogenously supplied growth factors, providing a basis for growth factor-reduced culture systems and cell-free strategies. Together, these findings illustrate that ribosome-induced cellular states function as a versatile platform linking cellular reprogramming to sustainable biotechnological applications.

To date, we have developed a technique utilizing ribosomes to confer pluripotency upon cells such as those from mice (Figure 2) [39]. As ribosomes include proteins universally present in life, they can be readily extracted from food ingredients. For instance, extracting ribosomes from fermentative microorganisms with established food safety records, such as lactic acid bacteria produced in large quantities as food ingredients, could potentially facilitate the scaling up of cultured cell food production by ensuring consistent cell quality. To explore applications for cellular food, we have demonstrated that ribosome uptake and pluripotency induction are also feasible in chicken-derived cells [47]. We believe this approach can be applied to many animal-derived cells used in cell-cultured food. Similar to tests utilizing mice, chicken muscle-derived cells, having acquired pluripotency, can be induced to differentiate into adipocytes, osteoblasts, chondrocytes, and other cell types [47]. These results suggest that the ribosome-based method for acquiring pluripotency not only stabilizes cell quality but also enables the reuse of the cells themselves. We believe this technology has the potential to develop into a method that significantly reduces the number of animals slaughtered.

## 5. Conclusions and Future Perspectives

In this review, we have outlined not only the canonical structure and function of ribosomes as the machinery for protein synthesis, but also emerging non-canonical roles that have become increasingly evident in recent years. Through concepts such as the Specialized Ribosomes hypothesis, ribosome-associated stress responses, and extraribosomal functions of ribosomal components, we have organized accumulating evidence suggesting that ribosomes act as multifaceted regulatory factors within cells rather than as passive translational machines. We further discussed that incorporation of exogenous ribosomes can induce phenotypic conversion in terminally differentiated somatic cells, reconstructing them into a cellular state characterized not only by acquisition of differentiation potential but also by active secretion of multiple growth factors. These observations support the view that ribosomes constitute a molecular basis capable of modulating cellular state.

In addition, we reviewed prior studies on stem cell-conditioned medium and highlighted its broad application in regenerative medicine. While the biological activity of conditioned medium has traditionally been attributed to stem cell-intrinsic secretory capacity, evidence that exogenous ribosomes can induce comparable secretory activity in non-stem cells prompts reconsideration of this assumption. Specifically, these findings suggest that paracrine activity is not an immutable property of cell identity, but rather an inducible feature determined by cellular state. Integration of ribosome-induced cell state modulation with conditioned-medium-based strategies may therefore provide new opportunities for next-generation regenerative medicine and tissue engineering.

A particularly promising application lies in the field of cell-cultured foods. In the context of global population growth and the urgent need for sustainable protein sources, challenges such as the so-called protein crisis and the environmental burden of livestock production have drawn increasing attention. Although animal cell-based food production represents a potential solution, reliance on exogenously supplied growth factors and fetal bovine serum remains a major obstacle in terms of cost and safety. Ribosome-based approaches may enable construction of low-cost, serum-free, and self-sustaining culture systems by conferring secretory activity on the cells themselves. Importantly, ribosomes derived from food-grade microorganisms such as lactic acid bacteria may satisfy industrial requirements for non-genetically modified and food-compatible inputs, offering societal value in terms of scalability, stabilization of cell quality, and reduction in animal use. Moreover, the secretory activity and plastic cellular states induced by ribosome incorporation are highly compatible with cell-free approaches in regenerative medicine. Therapeutic strategies that exploit informational molecules—such as secreted factors and EVs—rather than transplanted cells have the potential to circumvent tumorigenic risk and ethical concerns associated with stem cell transplantation and may become a central paradigm in future regenerative medicine. If conditioned medium derived from ribosome-induced cellular states exhibits multiple bioactivities, including anti-inflammatory effects, promotion of angiogenesis, and enhancement of cell proliferation, it could achieve regenerative outcomes comparable to those reported for induced pluripotent stem cell- or mesenchymal stem cell-derived conditioned media. In this context, ribosomes may function not as agents that convert cells themselves into therapeutic entities, but rather as catalytic triggers that activate cells as factories for the production of therapeutic factors.

Nevertheless, several challenges remain to be addressed for practical implementation. These include elucidation of the molecular mechanisms underlying cellular responses to exogenous ribosomes, quantitative characterization of secreted factor composition and abundance, and establishment of robust safety frameworks to mitigate immunogenicity and contamination risks. Addressing these issues through integrated transcriptomic, proteomic, and bioengineering approaches will be essential.

More importantly, the concept of ribosome-mediated cell state regulation presented here invites reconsideration of how cell fate is understood in developmental biology. Rather than viewing differentiation as a unidirectional and irreversible process, these findings support a framework in which cell fate is interpreted as a dynamic and context-dependent cellular state that can be reconfigured without genetic modification. This perspective reinforces a developmental view of cell identity as a continuum rather than a fixed attribute and introduces the concept that functional outcomes can be altered through state control.

Finally, research centered on ribosomes as regulators of cellular state has the potential to develop into a highly interdisciplinary field spanning developmental biology, microbiology, cell engineering, regenerative medicine, food science, and bioprocess engineering. In particular, the finding that microbial translation machinery can modulate eukaryotic cellular function and fate expands our understanding of host–microbe interactions and may create new interfaces with microbiome research. Overall, advancing our understanding of ribosome-mediated cell state control is expected to contribute not only to fundamental insights into life processes but also to innovation in regenerative medicine and cell culture technologies.

## Data Availability

For the preparation of this review, no new data was created.
